# A potential association between the characteristics of the multi-organ microbiota and lymph node metastasis in cervical cancer

**DOI:** 10.3389/fcimb.2025.1639811

**Published:** 2026-01-20

**Authors:** Lan Peng, Shuhui Yu, Yan Dong, Haifeng Gu, Zheng Li, Conghui Ai, Lan Zhang, Xingrao Wu

**Affiliations:** 1 Key Laboratory of Radiation Oncology, Yunnan Cancer Hospital, The Third Affiliated Hospital of Kunming Medical University, Peking University Cancer Hospital Yunnan, Kunming, China; 2 Key Laboratory of Pathology, Yunnan Cancer Hospital, The Third Affiliated Hospital of Kunming Medical University, Peking University Cancer Hospital Yunnan, Kunming, China; 3 Department of Gynecology, State Key Laboratory of Oncology in South China, Guangdong Provincial Clinical Research Center for Cancer, Sun Yat-sen University Cancer Center, Guangzhou, China; 4 Key Laboratory of Gynecologic Oncology, Yunnan Cancer Hospital, The Third Affiliated Hospital of Kunming Medical University, Peking University Cancer Hospital Yunnan, Kunming, China; 5 Key Laboratory of Radiology, Yunnan Cancer Hospital, The Third Affiliated Hospital of Kunming Medical University, Peking University Cancer Hospital Yunnan, Kunming, China

**Keywords:** biomarker, cervical cancer, lymph node metastasis, microbiota, predictive models

## Abstract

**Introduction:**

Microbiota alterations at multiple sites are associated with cervical cancer (CC). However, it is unclear whether CC lymph node metastasis (LNM) is indeed associated with microbiota alterations, whether the microbiota is generally suitable for screening CC LNM-related taxa.

**Materials and methods:**

We performed 16S rDNA sequencing of samples from oral swabs, feces, urine, and vaginal secretions from CC patients to clarify microbiota characteristics of LNM group. And we constructed a LNM prediction model for CC based on specific flora at each site.

**Results:**

The α-diversity of the urinary microbiota (*P*_Sob_ = 0.0272, *P*_Pielou_ = 0.0278, *P*_Shannon_ = 0.0209 and *P*_Simpson_ = 0.0465) was reduced in the LNM group compared to the non-LNM group, and significant differences were observed in the structure of the gut (R² = 0.0266, *P* = 0.033) and urine (R² = 0.0379, *P* = 0.002) microbiota between the two groups. The establishment of a predictive model based on oral specific flora, including *Erysipelotrichaceae UCG-003* sp., *Eubacterium halli group*, and *Staphylococcus* has enabled the differentiation of CC lymph node status. The area under the ROC curve was 0.798. The Yoden index, sensitivity and specificity of this prediction model were 0.520, 57.9% and 94.1%, respectively.

**Conclusion:**

CC patients with LNM have significant microbiological changes at multiple sites. The predictive model based on oral bacteria can provide a noninvasive and simple method for assessing LNM in CC.

## Introduction

Cervical cancer (CC) has been identified as the most common malignant tumor of the female reproductive tract, ranking fourth in terms of female cancer incidence and mortality (Sung et al., 2021). In recent years, it has been characterized by an increasing trend, affecting a younger demographic, and poses a significant health and economic threat (Ferlay et al., 2021; Chen et al., 2022b). LNM has been reported to be the primary route of tumor metastasis and a pivotal factor in the staging of CC, which is associated with tumor recurrence, distant metastasis and poor prognosis (Kawada and Taketo, 2011; Ruengkhachorn et al., 2015; Yang et al., 2022). The 2018 International Federation of Gynecology and Obstetrics (FIGO) staging system for CC incorporated LNM in the staging classification (Lee and Atri, 2019). Surgery was the preferred treatment option for early-stage CC, while concurrent radiotherapy was recommended for patients with locally advanced and moderately advanced disease. Consequently, it’s essential to define the lymph node status of CC patients for staging and treatment.

The genetic material carried by microbiota in the human body was designated the human microbiome, and it was determined that this played a key role in the initiation, progression, metastasis, and drug resistance of malignant tumors (Turnbaugh et al., 2007; Lloyd-Price et al., 2017; Cullin et al., 2021). Recent studies have indicated that the microbiota can modulate LNM in malignancy. It has been established that *Prevotella intermedia* enhances colorectal cancer (CRC) cell migration and invasion through secreted proteins as well as direct cell contact. Furthermore, its coexistence with *Fusobacterium nucleatum* produces a synergistic effect on this ability and correlates with lymph node and distant metastasis (Lo et al., 2022). In the tumor-resident microbiota of lung squamous cell carcinoma patients with LNM, the presence of certain biomarkers, *Proteus* and *Bacteroides*, has been observed to be associated with gene sets, including Oxidative Phosphorylation, Myc Targets, and E2F Targets, which may promote LNM (Liang et al., 2023). It is noteworthy that patients exhibiting LNM from malignant neoplasias, including thyroid cancer and colorectal cancer, have demonstrated a distinctive signature microbiota profile, which serves as an accurate predictor of lymph node status (Yu et al., 2022; Luu et al., 2023; Yinhang et al., 2023). Liu et al. demonstrated that Gram-negative bacteria, including *Escherichia coli*, *Prevotella bivia*, and *Fusobacterium nucleatum*, have the capacity to promote lymph node metastasis through the LPS-TLR4-MAPK-EFNA1/EDN2 signaling pathways (Liu et al., 2025). Despite the growing number of studies examining the role of the microbiome in promoting tumor lymph node metastasis, the impact of the microbiome on LNM in patients with CC remains to be elucidated.

In this study, we collected oral, stool, urine and vaginal secretion samples from CC patients, analyzed the differences in the structure of multi-site flora in the CC with and without LNM group. Furthermore, we screened for multi-site flora associated with LNM. A prediction model of LNM in CC was established, which provided a new prediction target for LNM in CC and a simple and straightforward diagnostic method for LNM in clinics.

## Materials and methods

### Study participants and ethical statement

The study population comprised patients with CC who were admitted to the Third Affiliated Hospital of Kunming Medical University between December 2021 and July 2022. Patients who met the following criteria were included in the study: (i) Squamous cell carcinoma or adenocarcinom. (ii) 18–75 years. (iii) No other tumors. (iv) Have not received anti-tumor therapy. (v) No antibiotics, probiotics or other pharmaceutical agents taken in the month prior to biospecimen collection. Exclusion criteria encompassed physilogical diagnosis of cervical sarcoma, neuroendocrine carcinoma, peripheral schwannoma or malignant melanoma, and instances of vaginal lavage within 1 week or sexual intercourse within 48 hours.

The diagnosis of LNM was based on pathological or imaging evidence. The criteria for LNM on computed tomography (CT) and magnetic resonance imaging (MRI) included a lymph node short-axis diameter of ≥10mm and/or the presence of specific morphological features, such as central hypodensity, central necrosis, circumferential enhancement of margins, non-smooth margins, and fusion of lymph nodes in cluster (Devine et al., 2019).

### Microbiome sample collection and sequencing

Vaginal secretion samples were collected from the posterior vaginal vault during gynecological examinations. Patients self-collected morning clean midstream urine and feces samples. Oral samples were self-collected by repeatedly wiping the cheeks and sides of the tongue with sterile disposable cotton swabs. All samples were flash frozen in liquid nitrogen and transferred to a -80 °C freezer within 1 hour of collection.

The microbial DNA of the experimental samples was extracted using the HiPure Stool DNA Kits (Magen, Guangzhou, China). The diluted genomic DNA was then used as a template for the polymerase chain reaction (PCR), which amplified the 16S rDNA V3-V4 of the bacteria, using specific primers with barcode sequences (341F-CCTAYGRGRBGCASCAG and 806R-GGACTACNNGGGTATCTAAT). The PCR products were purified using the AxyPrep DNA Gel Extraction Kit (Axygen Biosciences, Union City, CA, USA). The data was subsequently quantified utilizing the ABI Step One Plus Real-Time PCR System (Life Technologies, Foster City, CA, USA). The purified amplification products were mixed in equal amounts, ligated to sequencing splices, and sequencing libraries were constructed. Subsequently, sequencing was conducted on the Illumina Novaseq 6000 sequencing platform in PE250 mode (Modi et al., 2021).

### Sequence data processing

The initial raw reads were filtered to obtain high-quality clean reads in accordance with the following criteria, implemented using FASTP (Chen et al., 2018) (version 0.18.0): 1) Reads containing more than 10% of unknown nucleotides (N) were excluded; 2) Reads with less than 50% bases with a quality (Q-value) greater than 20 were excluded; 3) Adapter contamination was removed. Pared end clean reads were merged as raw tags using FLASH (Magoč and Salzberg, 2011) (version 1.2.11) with a minimum overlap of 10 bp and mismatch error rates of 2%. Noisy sequences were filtered to obtain high-quality, clean sequences, with the following conditions applied: 1) Break the raw tags from the first low-quality base site where the number of bases in the continuous low-quality value reaches the set length. The default quality threshold is ≤3 and the default length is 3 bp. 2) Then, tags with a continuous high-quality base length below 75% of the tag length were filtered (Caporaso et al., 2010). The clean tags were clustered into operational taxonomic units (OTUs) of ≥ 97% similarity using UPARSE (Edgar, 2013) (version 9.2.64) algorithm. All chimeric tags were removed using UCHIME (Edgar et al., 2011) algorithm and finally obtained effective tags for further analysis. The tag sequence with highest abundance was selected as representative sequence within each cluster. The representative OTU sequences were classified into organisms by a naive Bayesian model using RDP classifier (Wang et al., 2007) (version 2.2) based on SILVA database (Pruesse et al., 2007) (version 138.1), with the confidence threshold value of 0.8.

### Statistical analysis

The abundance statistics were visualized using Krona (Ondov et al., 2011) (version 2.6) for each species classification. The α-diversity indices, including Sob index, Pielou index, Shannon index, and Simpson index, were analyzed using QIIME, and the α-diversity of the two groups was compared using the Wilcoxon tests. Principal co-ordinate analysis (PCoA) based on the Bray-Curtis distance between samples was performed using the R package ‘Vegan’ and combined with the Adonis test to measure differences in β-diversity between groups. The indicator value of each species in the group was compared and the biomarkers were screened for each group. The biomarkers were developed using linear discriminant analysis (LDA) effect size (LEfSe). Functional pathway analysis was conducted using PICRUSt2, based on the Kyoto Encyclopedia of Genes and Genomes (KEGG) database (https://www.genome.jp/kegg/) (Kanehisa and Goto, 2000; Kanehisa et al., 2025), on 16S rDNA gene sequencing data obtained from two groups.

The statistical software used was IMB SPSS 29.0. The prediction model for LNM in CC patients was developed through binary logistic regression analysis. The receiver operating characteristic (ROC) curve was plotted to evaluate the performance of potential biomarkers. *P* < 0.05 was identified statistically significant difference.

### Patient and public involvement

Patients or the public were not involved in the design, or conduct, or reporting, or dissemination plans of our research. The purpose of this study was to evaluate the multi-site flora characteristics of patients with CC and its potential relationship to LNM. Researchers and hospital staff will support this work.

## Results

### Clinical characteristics

A total of 72 CC patients were included in this study, among whom 34 CC patients without LNM and 38 patients with LNM. A statistically significant difference was observed only in the maximum diameter of the tumors between the two groups (*P* < 0.001) (**Table 1**).

**Table 1:** Baseline clinical characteristics of CC patients with LNM and without LNM.

Item	non-LNM (n=34)	LNM (n=38)	*P* value
Age, y(mean ± SD)	54.56±14.37	51.66±11.85	0.107
< 50 years old	12(35.3)	15(39.5)	0.715
≥ 50 years old	22(64.7)	23(60.5)	
Number of pregnancies, n(%)			
<3	15 (44.1)	9 (23.7)	0.064
≥3	19 (55.9)	26 (68.4)	
Unknown	0	3 (7.9)	
Number of births, n(%)			
<3	23 (67.6)	25 (65.8)	0.358
≥3	11 (32.3)	10 (26.3)	
Unknown	0	3 (7.9)	
Ethics, n(%)			
Han	26(76.5)	28(73.7)	0.785
Other	8(23.5)	10(26.3)	
Histology, n(%)			
Squamous Cell Carcinoma	29(85.3)	33 (86.8)	1.000
Adenocarcinoma	5(14.7)	5 (12.2)	
Tumor diameter, n(%)			
< 4cm	21 (61.8)	6(15.8)	<0.001
≥ 4cm	13 (38.2)	32(84.2)	

### Microbial compositions correlate with lymph node status

**Figure 1:**
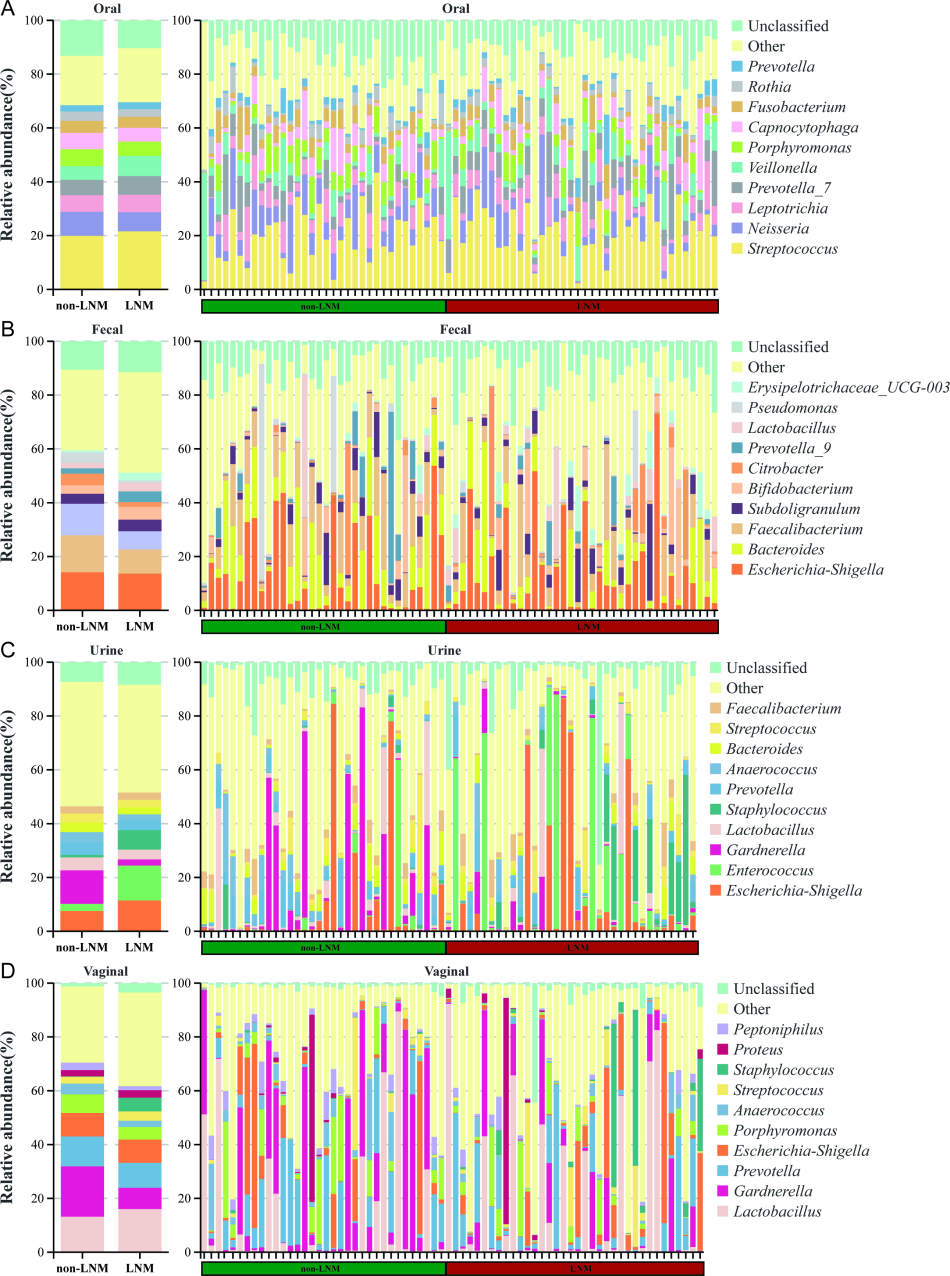
Genus-level microbiota composition of multiple sites in cervical cancer patients with/without lymph node metastasis (LNM). **(A)** Oral microbiota. **(B)** Fecal microbiota. **(C)** Urine microbiota. **(D)** Vaginal microbiota. Stacked bar charts of species distribution at the genus level. These charts aim to intuitively display the composition and proportional relationship of microbiota species in different body sites between cervical cancer patients with LNM and without LNM, clearly reflecting the intergroup and intersample differences in species. The top 10 most abundant species in each site are presented individually, while other known species are merged into “Other” and unclassified species are labeled as “Unclassified”.

The predominant oral flora in CC patients was identified as *Streptococcus* (**Figure 1A**). In the gut microbiota of CC patients, the relative abundance of *Bacteroides* (*P* < 0.046) and *Fecalibacterium* (*P* < 0.002) was found to be reduced in the LNM group in comparison to the non-LNM group (**Figure 1B**). Within the urethra, *Enterococcus* (*P* < 0.004) demonstrated an increase in relative abundance in comparison to the non-LNM group, whereas *Gardnerella* (*P* < 0.038), *Lactobacillus* (*P* < 0.002) and *Anaerococcus* (*P* < 0.004) exhibited significantly lower relative abundance than the CC without LNM (**Figure 1C**). The relative abundance of *Gardnerella* (*P* < 0.012) and *Peptoniphilus* (*P* < 0.038) of vaginal flora was found to be significantly lower in the LNM group compared to the non-LNM group (**Figure 1D**).

### Microbial diversity of CC patients with and without LNM

**Figure 2:**
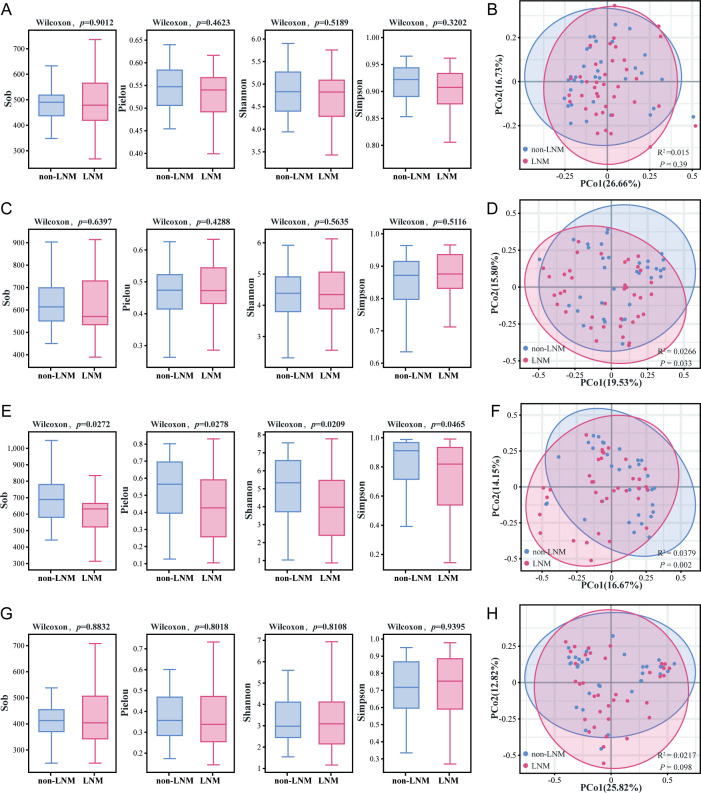
Biodiversity analysis of the microbial community in cervical cancer patients with vs. without LNM. **(A, B)** Oral microbiota. **(C, D)** Fecal microbiota. **(E, F)** Urine microbiota. **(G, H)** Vaginal microbiota. α-diversity (Left): Sobs, Pielou, Simpson, and Shannon indices were used to evaluate species richness and evenness, with intergroup differences compared by the Wilcoxon test (*P* < 0.05). β-diversity (Right): Principal Co-ordinate Analysis (PCoA) was performed to visualize differences in community structure between groups, and Adonis test was used to quantify the statistical significance of intergroup β-diversity differences (*P* < 0.05).

There were no significant differences in the α-diversity (*P*_Sob_ = 0.9012, *P*_Pielou_ = 0.4623, *P*_Shannon_ = 0.5189 and *P*_Simpson_ = 0.3202) and β-diversity (R² = 0.015, *P* = 0.39) of oral microbiota between the two groups (**Figures 2A, B**, **Supplementary Table 1**). No notable discrepancy was observed in the α-diversity (*P*_Sob_ = 0.6397, *P*_Pielou_ = 0.4288, *P*_Shannon_ = 0.5635 and *P*_Simpson_ = 0.5116) of fecal microbiota between the two cohorts of CC patients (**Figure 2C**, **Supplementary Table 1**). However, a substantial divergence was evident in β-diversity (R^2^ = 0.0266, *P* = 0.033)(**Figure 2D**). The urinary microbiota of CC patients in the LNM group exhibited reduced species richness and uniformity compared to the non-LNM group (*P*_Sob_ = 0.0272, *P*_Pielou_= 0.0278, *P*_Shannon_ = 0.0209 and *P*_Simpson_ = 0.0465) (**Figure 2E**, **Supplementary Table 1**), and there were significant differences in the microbial composition (R^2^ = 0.0379, *P* = 0.002) (**Figure 2F**). Moreover, no significant divergence was evident in the α-diversity (*P*_Sob_ = 0.8832, *P*_Pielou_ = 0.8018, *P*_Shannon_= 0.8018 and *P*_Simpson_ = 0.9395) and β-diversity (R^2^ = 0.0217, *P* = 0.098) of the vaginal microbiota between the two cohorts (**Figures 2G, H**, **Supplementary Table 1**).

### Screening of microbial biomarkers in CC patients with LNM and establishment of a clinical predictive model

**Figure 3:**
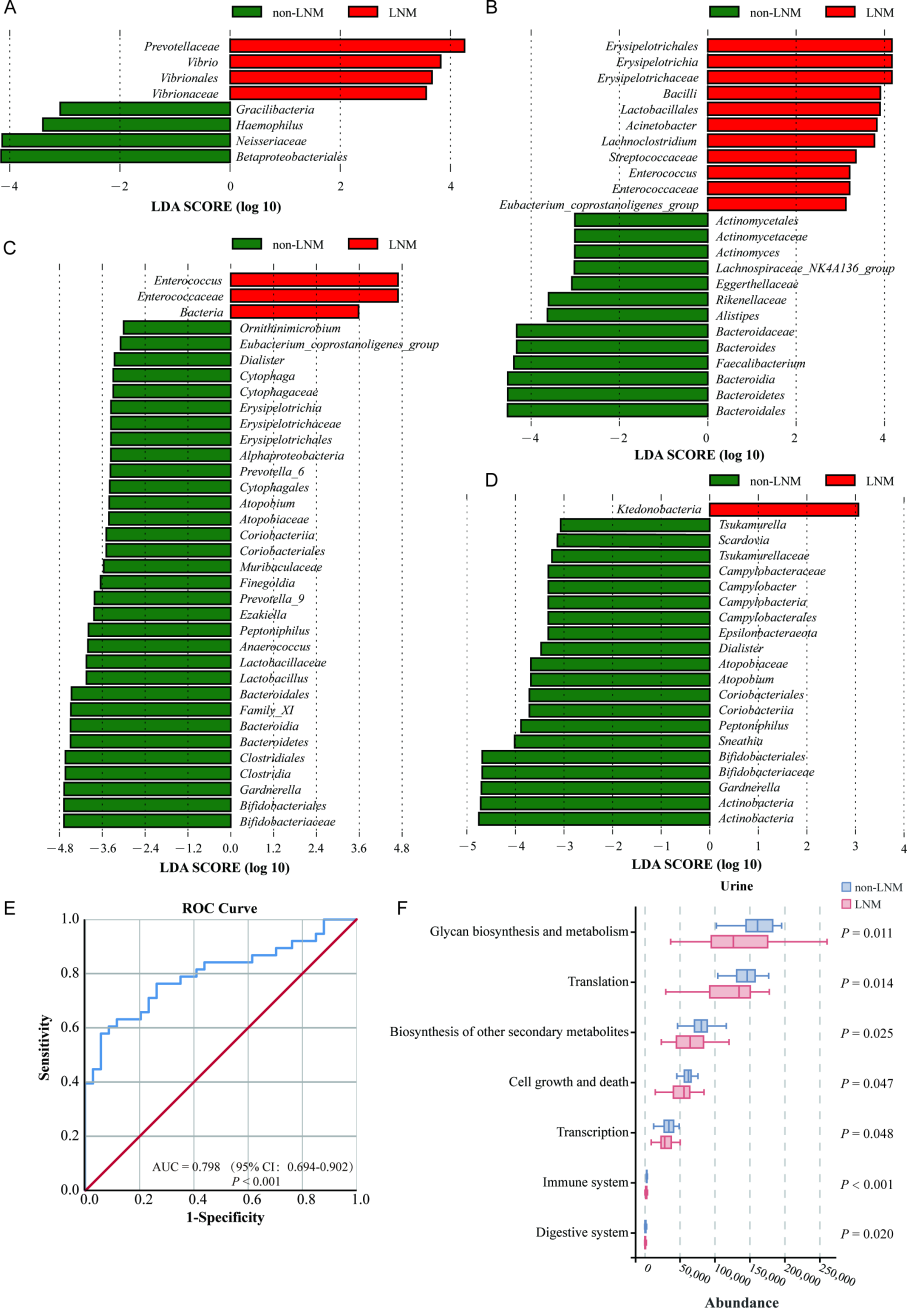
Development of a LNM prediction model in cervical cancer patients. Linear discriminant analysis effect size (LEfSe) analysis identified differential microbial biomarker, which were then visualized by LDA bar charts. The length of the bar is indicative of the impact strength of each differential species (corresponding to LDA Score), thus distinguishing characteristic species between groups. **(A-D)** LEfSe analysis was performed to identify microbial biomarkers of the oral **(A)**, fecal **(B)**, urinary **(C)**, and vaginal **(D)** microbiota in CC patients with vs. without LNM (LDA score > 3, *P* < 0.05). **(E)** Receiver Operating Characteristic (ROC) curve was used to evaluate the predictive performance of the model for LNM risk. The Area Under the Curve (AUC) is a core indicator of model performance, with values corresponding to predictive accuracy levels: low accuracy (AUC = 0.5–0.7), moderate accuracy (AUC = 0.7–0.9), and high accuracy (AUC>0.9). **(F)** Functional prediction analysis of the urinary microbiota to compare differential functions between CC patients with and without LNM.

The results of the indicator analysis demonstrated that *Atopobium*, *Acinetobacter*, *Bifidobacterium*, *Eubacterium hallii group*, *Erysipelotrichaceae UCG-003* sp., *Ruminococcus torques group* and *Staphylococcus* were identified as oral microbial biomarkers of the LNM group (**Supplementary Table 2**). A total of nine indicator genera were identified within the gut microbiota of the LNM group, and eight biomarkers were screened from the urine flora (**Supplementary Tables 3, 4**). The marker genera for the vaginal flora of the LNM group were *Staphylococcus*, *Enterococcus*, *Erysipelotrichaceae UCG-003* sp., *Lysinibacillus* and *Psychrobacter* (**Supplementary Table 5**). At the genus level, the LEfSe analysis revealed that the biomarkers of oral flora in the LNM group was *Vibrio* (**Figure 3A**, **Supplementary Figure 1A**). Four intestinal flora, *Acinetobacter*, *Lachnoclostridium*, *Enterococcus* and *Eubacterium coprostanoligenes group*, were identified as biomarkers for CC in the gut microbiota of the LNM group (**Figure 3B**, **Supplementary Figure 1B**). In the urinary microbiota, only *Enterococcus* was enriched in the LNM group (**Figure 3C**, **Supplementary Figure 1C**). No statistically significant marker flora was identified in the vaginal microbiota of the LNM group (**Figure 3D**, **Supplementary Figure 1D**).

The multi-site marker genera of LNM, as identified through LEfSe analysis and indicator analysis, were subjected to establish predictive models. The relative abundance of the oral microbiota, *Staphylococcus*, *Erysipelotrichaceae UCG-003* sp. and *Eubacterium hallii group*, was identified as an independent influencing factor for the occurrence of LNM in patients with CC (*P* < 0.05) (**Supplementary Table 6**). The predictive model was calculated:

logit (*P*) = -1.344 + 52.081 × *Staphylococcus* + 320.699 × *Erysipelotrichaceae UCG -003* sp.- 237.932 × *Eubacteriu halli group*.

The prediction model predicted LNM in CC patients with high accuracy (AUC = 79.8%) (**Figure 3E**). The model demonstrated a Yoden index of 0.520 and a Logit (*P*) value exceeding 0.692, which corresponded to the cut-off value, thereby providing a risk stratification criterion for clinical practice. This resulted in a sensitivity of 57.9% and a specificity of 94.1%, enabling effective screening of patients at low risk of LNM. The Hosmer-Lemeshow *χ^2^* statistic was 3.030 (*P* = 0.932 > 0.05), indicating excellent model calibration and reliable clinical risk prediction.

### Altered pathways in multi-site microbial communities of CC patients with LNM

Subsequently, the functional and metabolism-related alterations in the microbiota at multiple sites in CC patients with LNM were analyzed. A statistically significant difference in KEGG pathways was observed only in the urinary microbiota between the two groups. Seven KEGG pathways exhibited significant differences between the two groups, including glycan biosynthesis and metabolism (*P* = 0.011), biosynthesis of other secondary metabolites (*P* = 0.025), transcription (*P* = 0.048), translation (*P* = 0.014), Cell growth and death (*P* = 0.047), immune system (*P* < 0.001) and digestive system (*P* = 0.020) (**Figure 3F**).

## Discussion

We have previously reported the characteristics of the microbiome of multiple mucosal organs in patients with CC (Peng et al., 2024). Herein, this study is pioneering in its investigation of a potential correlation between multi-organ microbiome and the metastatic status of lymph nodes in CC patients. The data revealed alterations in the relative abundance and composition of the microbiota from multiple sites in LNM group, and a prediction model for determining LNM status in CC patients was constructed based on specific oral flora.

The female vaginal cavity is an open, externally-contacted anatomical structure that is vital for host-microbe interactions. *Lactobacillus* maintains the normal acidic environment of the vagina and prevents infection by sexually transmitted pathogens and opportunistic infections (Ravel et al., 2011; Miller et al., 2016). It has been shown that *Lactobacillus* can be incorporated into dense biofilms formed by pathogens, thereby producing a disruptive and killing effect and reducing the incidence and recurrence of inflammatory diseases in the reproductive tract (McMillan et al., 2011). *Lactobacillus* has been observed to induce interferon-γ production by human peripheral blood mononuclear cells, which assist immune cells in clearing human papillomavirus (HPV) infections, thereby promoting immune homeostasis (Nicolò et al., 2021). Lactic acid, a metabolite of *Lactobacillus*, activated the Wnt pathway via the lactate-Gpr81 complex, which increased the level of core fucosylation of epithelial cells and inhibited the proliferation and migration of CC cells (Fan et al., 2021). The relationship between vaginal flora imbalance and the development of CC has been the subject of further research. As low-grade squamous intraepithelial lesions progress to high-grade squamous intraepithelial lesions and invasive carcinoma of the cervix, the diversity of the vaginal flora gradually increases, the abundance of *Lactobacillus* continues to decrease, and multiple pathogenic bacteria, including *Gardnerella* and *Prevotella*, increase dramatically (Mitra et al., 2015; Liu et al., 2022). It is currently thought that vaginal dysbiosis may induce CC through a number of potential mechanisms, including the disruption of the integrity of the vaginal mucosa and epithelium, the mediation of HPV infection, the creation of a pro-inflammatory environment that promotes the integration of HPV DNA, and the influence of immunomodulation (Mitra et al., 2016; Champer et al., 2018).

The gastrointestinal tract serves as the primary habitat for the human microbiota, which contains a vast number of gut bacteria, estimated in the trillions (Sender et al., 2016). The existence of bidirectional communication between the vaginal and gut microbiota has been demonstrated, with the gut flora potentially functioning as a reservoir for vaginal pathogens (Kyrgiou and Moscicki, 2022). The gut microbiome differed between CC patients and healthy women. Bacterial species such as *Parabacteroides*, *Escherichia-Shigelle*, *Roseburia*, *Prevotella*, *Porphyromonas* and *Dialister* were enriched in CC (Sims et al., 2019; Wang et al., 2019). A study by Chang et al. revealed that the intestinal colonies of CC patients exhibited structural and functional differences from those of healthy women. Additionally, the study identified a negative correlation between the stage of CC and the presence of *Ruminococcus*, which could be taken as a clinically relevant biomarker (Chang et al., 2023). An imbalance in the intestinal flora has been linked to tumor development and progression through various mechanisms, including regulation of estrogen levels, modulation of inflammatory responses, interference with carbohydrate metabolism, and the production of toxins and metabolites by dysbiosis of the intestinal flora (Łaniewski et al., 2020).

This study predicted the LNM status of CC based on the marker genus at each site, and found that *Staphylococcus*, *Erysipelotrichaceae UCG-003* sp., and *Eubacterium halli group* of oral flora were independent influencing factors on LNM in CC patients. SNases are extracellular nucleases of Staphylococcus aureus. The oncoprotein SND1, which is a human homologue of the SNase, has been demonstrated to be closely associated with the development and progression of several cancer (Wei et al., 2022). Ma et al. found that HPV-16 was invasive in Enterococcus and Staphylococcus, and that the HPV-16 E7 and L1 genes could be transcribed in bacterial cells, thereby facilitating persistent HPV-16 infection and the development of CC (Ma et al., 2009). The relative abundance of *Erysipelotrichaceae UCG-003* sp. in the gut microbiome of lung cancer patients was significantly lower than that of the healthy population and negatively correlated with glycerophospholipids metabolism, which is involved in regulating metabolism and tumor development (Zhao et al., 2021). The *Eubacterium halli group* was identified as the predominant gut flora in patients with renal clear cell carcinoma (Chen et al., 2022a). *Eubacterium halli group* in the gut of elderly patients with liver cancer was negatively correlated with the levels of liver enzymes (Zhang et al., 2023). However, studies about the role of *Erysipelotrichaceae UCG-003* sp. and the *Eubacterium halli group* in cervical carcinogenesis and progression are currently lacking.

Recent studies have elucidated the mechanism through which the microbiome contributes to tumor metastasis. Fu et al. demonstrated that the invasion of tumor cells by specific intratumoral flora remodeled the cytoskeleton via the RhoA-ROCK pathway. This process increased the tolerance of circulating tumor cells to intravascular mechanical stress, reduced cell death during metastasis, and promoted distant metastasis of tumor cells (Fu et al., 2022). Bullman et al. revealed that the bacterial profiles of primary foci and metastatic tumors in colorectal cancer patients are highly consistent and that the microorganisms metastasize along with the tumor cells (Bullman et al., 2017). Parida et al. demonstrated that enterotoxigenic *Bacteroides fragilis* promoted the aggressive progression and metastasis of breast cancer (Parida et al., 2021). *Fusobacterium nucleatum* within colorectal cancer tumors survived and proliferated in macrophages, inducing host tumor cells to produce a substantial quantity of exosomes enriched for miR-1246/92b-3p/27a-3p and CXCL16/RhoA/IL-8, which promote tumor metastasis (Guo et al., 2020). *Fusobacterium nucleatum* induced a decrease in m6A modification through the YAP/FOXD3/METTL3 pathway, which led to the up-regulation of KIF26B and contributed to the enhancement of colorectal cancer cell invasiveness (Chen et al., 2022).

This study showed that bacterial dysbiosis in multiple sites of CC patients were associated with the occurrence of LNM. The oral microbial biomarkers, *Staphylococcus*, *Erysipelotrichaceae UCG-003* sp. and *Eubacterium hallii group*, offer the advantage of non-invasive detection, rendering them suitable for large-scale CC with LNM screening and follow-up. The integration of microbiomics into the CC diagnosis and treatment system holds considerable potential for enhancing screening accuracy, optimizing treatment regimens, and improving patient prognosis. This integration would also pioneer new avenues for precision medicine in CC.

Nevertheless, it should be acknowledged that the study is not without its limitations. Firstly, it is acknowledged that persistent HPV infection constitutes the primary causative factor for CC, and considering the absence of HPV infection history records for some patients in this study, HPV information was excluded from the analysis to mitigate risks of data bias. This may constrain the clinical applicability of the target microbiome and potentially obscure differences in biomarker predictive efficacy between populations with varying HPV infection statuses. Secondly, it has been shown that diet type have the capacity to disrupt the microbiota, thereby influencing tumor initiation and progression (Fu et al., 2019; Fu et al., 2023; Nakatsu et al., 2024). The absence of diet type information may compromise the generalizability of predictive models. Thirdly, the study is limited by its single-center design and small sample size, resulting in poor external validity and model stability. In order to validate the predictive value of the model, further expansion is required.

## Conclusions

Our findings characterize the dysbiosis of microbiota at multi-site in CC patients with LNM. The predictive model constructed based on oral-specific flora was able to distinguish CC patients with LNM from those without LNM, thus providing new diagnostic ideas for clinical work, new ideas and insights for personalized treatment of CC, and an important resource for researchers conducting studies in this field.

## Data Availability

The original contributions presented in the study are included in the article/**Supplementary Material**. Further inquiries can be directed to the corresponding authors.

## Supplementary material

Supplementary Figure 1 LEfSe analysis identified differential microbial biomarkers visualized by Taxonomic cladogram (LDA score > 3, *P* < 0.05). The innermost to outermost radiating circles correspond to taxonomic levels ranging from kingdom to species, with only the genus level displayed in this study. Each small circle at a given taxonomic level represents a species within that level, and the diameter of the small circle is positively correlated with the relative abundance of the species (larger diameter indicates higher relative abundance).
