# Supplementary figures and images for: A potential association between the characteristics of the multi-organ microbiota and lymph node metastasis in cervical cancer
Source: Front Cell Infect Microbiol. 2026 Jan 20;15:1639811. doi: 10.3389/fcimb.2025.1639811 (PMC12864395; doi:10.3389/fcimb.2025.1639811)

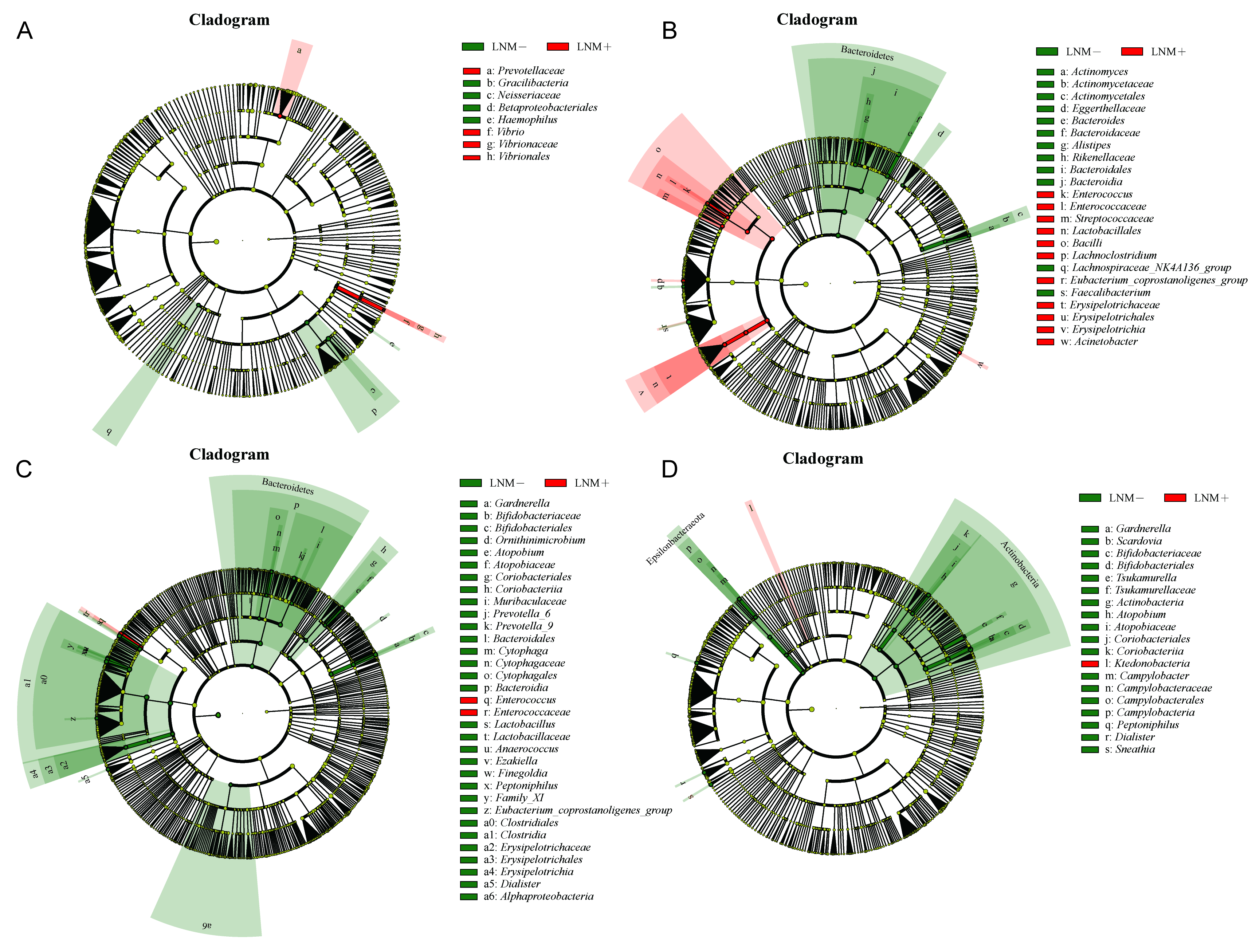

Supplement: Supplementary Figure 1 — LEfSe analysis identified differential microbial biomarkers visualized by Taxonomic cladogram (LDA score > 3, P < 0.05). The innermost to outermost radiating circles correspond to taxonomic levels ranging from kingdom to species, with only the genus level displayed in this study. Each small circle at a given taxonomic level represents a species within that level, and the diameter of the small circle is positively correlated with the relative abundance of the species (larger diameter indicates higher relative abundance). [file Image1.tif]
