# Supplementary material for: A potential association between the characteristics of the multi-organ microbiota and lymph node metastasis in cervical cancer
Source: Front Cell Infect Microbiol. 2026 Jan 20;15:1639811. doi: 10.3389/fcimb.2025.1639811 (PMC12864395; doi:10.3389/fcimb.2025.1639811)
Supplement: Supplementary file 2 [file Table1.docx]

**Supplementary Table 1** Multi-site microbiome α-diversity index in CC patients with and without LNM

| Site | Group | Sob index | *P* value | Pielou index | *P* value | Shannon index | *P* value | Simpson index | *P* value |
| --- | --- | --- | --- | --- | --- | --- | --- | --- | --- |
| Oral | non-LNM | 480.29412 | 0.9012 | 0.541013 | 0.4623 | 4.817482 | 0.5189 | 0.909638 | 0.3202 |
|  | LNM | 491.44737 |  | 0.529707 |  | 4.724203 |  | 0.900141 |  |
| Fecal | non-LNM | 631.97059 | 0.6397 | 0.458837 | 0.4288 | 4.271318 | 0.5635 | 0.839283 | 0.5116 |
|  | LNM | 619.81579 |  | 0.475161 |  | 4.410937 |  | 0.855798 |  |
| Urine | non-LNM | 694.23529 | **0.0272** | 0.524712 | **0.0278** | 4.954331 | **0.0209** | 0.806639 | **0.0465** |
|  | LNM | 591.57143 |  | 0.428511 |  | 3.944707 |  | 0.717101 |  |
| Vaginal | non-LNM | 421.52941 | 0.8832 | 0.367528 | 0.8018 | 3.206453 | 0.8108 | 0.724722 | 0.9395 |
|  | LNM | 437.44444 |  | 0.367499 |  | 3.225709 |  | 0.712244 |  |

**Supplementary Table 2** Screening of oral flora marker genus in two groups of CC patients by Indictor analysis

| Group | Genus | Indicator value | *P* value |
| --- | --- | --- | --- |
| LNM | *Atopobium* | 0.71801 | 0.019 |
| LNM | *Acinetobacter* | 0.83046 | 0.032 |
| LNM | *Staphylococcus* | 0.85503 | 0.001 |
| LNM | *Bifidobacterium* | 0.58063 | 0.034 |
| LNM | *Erysipelotrichaceae_UCG-003* | 0.53782 | 0.015 |
| LNM | *Ruminococcus_torques_group* | 0.56981 | 0.011 |
| LNM | *Eubacterium_hallii_group* | 0.49353 | 0.041 |
| non-LNM | *Bergeyella* | 0.63851 | 0.028 |

**Supplementary Table 3** Screening of gut flora marker genus in two groups of CC patients by Indictor analysis

| Group | Genus | Indicator value | P value |
| --- | --- | --- | --- |
| LNM | *Erysipelotrichaceae_UCG-003* | 0.78092 | 0.006 |
| LNM | *Lachnoclostridium* | 0.70094 | 0.001 |
| LNM | *Eubacterium_hallii_group* | 0.68895 | 0.038 |
| LNM | *Ruminococcus_gnavus_group* | 0.72730 | 0.047 |
| LNM | *Hungatella* | 0.85105 | 0.045 |
| LNM | *Staphylococcus* | 0.68230 | 0.013 |
| LNM | *Anaerococcus* | 0.62659 | 0.05 |
| LNM | *Senegalimassilia* | 0.47676 | 0.049 |
| LNM | *Chryseobacterium* | 0.51117 | 0.032 |
| non-LNM | *Faecalibacterium* | 0.63367 | 0.025 |
| non-LNM | *Tyzzerella_3* | 0.88721 | 0.025 |
| non-LNM | *Ruminococcus_1* | 0.72603 | 0.027 |
| non-LNM | *Lachnospiraceae_NK4A136_group* | 0.78514 | 0.011 |
| non-LNM | *Negativibacillus* | 0.47933 | 0.041 |

**Supplementary Table 4** Screening of urine flora marker genus in two groups of CC patients by Indictor analysis

| Group | Genus | Indicator value | P value |
| --- | --- | --- | --- |
| LNM | *Enterococcus* | 0.82994 | 0.023 |
| LNM | *Staphylococcus* | 0.88619 | 0.007 |
| LNM | *Leptotrichia* | 0.81359 | 0.03 |
| LNM | *Lautropia* | 0.70042 | 0.017 |
| LNM | *Abiotrophia* | 0.57295 | 0.048 |
| LNM | *Psychrobacillus* | 0.36558 | 0.041 |
| LNM | *Selenomonas_3* | 0.41705 | 0.043 |
| LNM | *Johnsonella* | 0.42643 | 0.01 |
| non-LNM | *Gardnerella* | 0.84528 | 0.015 |
| non-LNM | *Ezakiella* | 0.75153 | 0.023 |
| non-LNM | *Prevotella_6* | 0.71727 | 0.03 |
| non-LNM | *Eubacterium_coprostanoligenes_group* | 0.71156 | 0.015 |
| non-LNM | *Cytophaga* | 0.55620 | 0.001 |
| non-LNM | *Parabacteroides* | 0.72714 | 0.013 |
| non-LNM | *Megamonas* | 0.68124 | 0.034 |
| non-LNM | *Ruminococcaceae_UCG-002* | 0.76312 | 0.006 |
| non-LNM | *Rikenellaceae_RC9_gut_group* | 0.64887 | 0.047 |
| non-LNM | *Ruminococcaceae_UCG-005* | 0.66621 | 0.022 |
| non-LNM | *Christensenellaceae_R-7_group* | 0.71074 | 0.03 |
| non-LNM | *Ruminococcus_gnavus_group* | 0.78647 | 0.007 |
| non-LNM | *Mobiluncus* | 0.69471 | 0.003 |
| non-LNM | *Phascolarctobacterium* | 0.72123 | 0.006 |
| non-LNM | *Romboutsia* | 0.65494 | 0.026 |
| non-LNM | *Varibaculum* | 0.73441 | 0.007 |
| non-LNM | *Dubosiella* | 0.70130 | 0.005 |
| non-LNM | *Aerococcus* | 0.78377 | 0.002 |
| non-LNM | *Lachnospira* | 0.69701 | 0.016 |
| non-LNM | *Eubacterium_eligens_group* | 0.65582 | 0.047 |
| non-LNM | *S5-A14a* | 0.61025 | 0.012 |
| non-LNM | *Ruminococcaceae_NK4A214_group* | 0.66584 | 0.022 |
| non-LNM | *Treponema_2* | 0.60454 | 0.042 |
| non-LNM | *Asticcacaulis* | 0.49398 | 0.027 |
| non-LNM | *Prevotellaceae_UCG-001* | 0.55836 | 0.049 |
| non-LNM | *Ruminococcaceae_UCG-010* | 0.67366 | 0.006 |
| non-LNM | *Prevotellaceae_NK3B31_group* | 0.72954 | 0.001 |
| non-LNM | *Butyricimonas* | 0.59917 | 0.018 |
| non-LNM | *Lachnospiraceae_ND3007_group* | 0.73474 | 0.003 |
| non-LNM | *Catenibacterium* | 0.68087 | 0.005 |
| non-LNM | *Dyadobacter* | 0.52692 | 0.001 |
| non-LNM | *Family_XIII_AD3011_group* | 0.64806 | 0.011 |
| non-LNM | *W5053* | 0.57225 | 0.027 |
| non-LNM | *Methanobrevibacter* | 0.53432 | 0.003 |
| non-LNM | *Prevotellaceae_UCG-003* | 0.47049 | 0.004 |
| non-LNM | *Gallicola* | 0.52851 | 0.031 |
| non-LNM | *Muribaculum* | 0.51793 | 0.005 |
| non-LNM | *Holdemanella* | 0.66835 | 0.014 |
| non-LNM | *Lachnospiraceae_XPB1014_group* | 0.54255 | 0.001 |
| non-LNM | *Bilophila* | 0.69217 | 0.011 |
| non-LNM | *Cellvibrio* | 0.42467 | 0.019 |
| non-LNM | *Lysinibacillus* | 0.57460 | 0.016 |
| non-LNM | *Massilia* | 0.60869 | 0.01 |
| non-LNM | *Larkinella* | 0.35145 | 0.021 |
| non-LNM | *dgA-11_gut_group* | 0.37163 | 0.005 |
| non-LNM | *Psychrobacter* | 0.62858 | 0.009 |
| non-LNM | *Eubacterium_fissicatena_group* | 0.61256 | 0.014 |
| non-LNM | *Blastomonas* | 0.43669 | 0.008 |
| non-LNM | *Slackia* | 0.62369 | 0.018 |
| non-LNM | *Cloacibacillus* | 0.52445 | 0.001 |
| non-LNM | *Skermanella* | 0.43737 | 0.001 |
| non-LNM | *Geobacillus* | 0.43036 | 0.025 |
| non-LNM | *Ruminococcaceae_UCG-009* | 0.49381 | 0.004 |
| non-LNM | *Fournierella* | 0.31717 | 0.02 |
| non-LNM | *Adlercreutzia* | 0.43462 | 0.041 |
| non-LNM | *Thermomonas* | 0.34626 | 0.015 |
| non-LNM | *Anoxybacillus* | 0.43017 | 0.001 |
| non-LNM | *Ruminococcaceae_UCG-008* | 0.43737 | 0.017 |
| non-LNM | *Propionimicrobium* | 0.34377 | 0.04 |
| non-LNM | *Mucispirillum* | 0.37789 | 0.014 |
| non-LNM | *Rikenella* | 0.39539 | 0.01 |
| non-LNM | *Anaerorhabdus_furcosa_group* | 0.33226 | 0.004 |
| non-LNM | *p-1088-a5_gut_group* | 0.29523 | 0.017 |
| non-LNM | *Pseudoxanthomonas* | 0.26426 | 0.004 |
| non-LNM | *Singulisphaera* | 0.23432 | 0.034 |
| non-LNM | *Ochrobactrum* | 0.37803 | 0.033 |
| non-LNM | *Coprobacter* | 0.40185 | 0.044 |
| non-LNM | *Hymenobacter* | 0.28950 | 0.03 |
| non-LNM | *Synergistes* | 0.29151 | 0.017 |
| non-LNM | *Sphingoaurantiacus* | 0.29041 | 0.017 |
| non-LNM | *CAG-352* | 0.20415 | 0.018 |
| non-LNM | *Novosphingobium* | 0.23427 | 0.01 |
| non-LNM | *Saccharopolyspora* | 0.20427 | 0.022 |

**Supplementary Table 5** Screening of vaginal flora marker genus in two groups of CC patients by Indictor analysis

| Group | Genus | Indicator value | P value |
| --- | --- | --- | --- |
| LNM | *Staphylococcus* | 0.941004 | 0.016 |
| LNM | *Enterococcus* | 0.920006 | 0.006 |
| LNM | *Erysipelotrichaceae_UCG-003* | 0.581843 | 0.045 |
| LNM | *Lysinibacillus* | 0.303688 | 0.023 |
| LNM | *Psychrobacter* | 0.333733 | 0.045 |
| non-LNM | *Gardnerella* | 0.7029 | 0.023 |
| non-LNM | *Atopobium* | 0.85899 | 0.024 |
| non-LNM | *Aerococcus* | 0.912907 | 0.01 |
| non-LNM | *DNF00809* | 0.708927 | 0.008 |
| non-LNM | *Arcanobacterium* | 0.610633 | 0.026 |
| non-LNM | *Varibaculum* | 0.64225 | 0.005 |

**Supplementary Table 6** Binary logistic regression analysis of oral flora marker genus

| Genus | B | SE | Wald | *P* value | OR | 95%CI |
| --- | --- | --- | --- | --- | --- | --- |
|  | -1.344 | 0.471 | 8.146 | 0.004 | 0.261 | - |
| *Atopobium* | 2.631 | 1.439 | 3.344 | 0.067 | 13.889 | 0.828~233.047 |
| *Acinetobacter* | 2.917 | 2.715 | 1.154 | 0.283 | 18.479 | 0.090~3781.534 |
| *Staphylococcus* | 52.081 | 25.856 | 4.057 | **0.044** | 4.1541×10^22^ | 4.075~4.235×10^44^ |
| *Bifidobacterium* | 4.236 | 26.102 | 0.026 | 0.871 | 69.106 | 0.000~1.142×10^24^ |
| *Erysipelotrichaceae_UCG-003* | 320.699 | 138.532 | 5.359 | **0.021** | 1.896×10^139^ | 2.288×10^21^~1.571×10^257^ |
| *Ruminococcus_torques_group* | -69.655 | 102.113 | 0.465 | 0.495 | .000 | 0.000~4.650×10^56^ |
| *Eubacterium_hallii_group* | -237.932 | 115.861 | 4.217 | **0.040** | .000 | 0.000~0.000 |
